# Responsive and Equitable Health Systems—Partnership on Non-Communicable Diseases (RESPOND) study: a mixed-methods, longitudinal, observational study on treatment seeking for hypertension in Malaysia and the Philippines

**DOI:** 10.1136/bmjopen-2018-024000

**Published:** 2018-07-30

**Authors:** Benjamin Palafox, Maureen L Seguin, Martin McKee, Antonio L Dans, Khalid Yusoff, Christine J Candari, Khairuddin Idris, Johan Rizwal Ismail, Steven Eric Krauss, Gideon Lasco, Fadhlina A Majid, Lia M Palileo-Villanueva, Azlina A Razak, Alicia Renedo, Dina Balabanova

**Affiliations:** 1 Centre for Global Chronic Conditions, London School of Hygiene and Tropical Medicine, London, UK; 2 College of Medicine, University of the Philippines Manila, Manila, Philippines; 3 Faculty of Medicine and Health Sciences, UCSI University, Kuala Lumpur, Malaysia; 4 Centre for Translational Research and Epidemiology, Faculty of Medicine, University Teknologi MARA, Shah Alam, Malaysia; 5 Institute for Social Science Studies, University Putra Malaysia, Serdang, Malaysia; 6 Department of Anthropology, University of the Philippines Diliman, Quezon City, Philippines; 7 Social and Environmental Health Research, London School of Hygiene and Tropical Medicine, London, UK

**Keywords:** hypertension, health systems, cardiovascular disease, malaysia, philippines

## Abstract

**Introduction:**

Hypertension is a leading contributor to the global burden of disease. While safe and effective treatment exists, blood pressure control is poor in many countries, often reflecting barriers at the levels of health systems and services as well as at the broader level of patients’ sociocultural contexts. This study examines how these interact to facilitate or hinder hypertension control, taking into account characteristics of service provision components and social contexts.

**Methods and analysis:**

The study, set in Malaysia and the Philippines, builds on two systematic reviews of barriers to effective hypertension management. People with hypertension (pre-existing and newly diagnosed) will be identified in poor households in 24–30 communities per country. Quantitative and qualitative methods will be used to examine their experiences of and pathways into seeking and obtaining care. These include two waves of household surveys of 20–25 participants per community 12–18 months apart, microcosting exercises to assess the cost of illness (including costs due to health seeking activities and inability to work (5 per community)), preliminary and follow-up in-depth interviews and digital diaries with hypertensive adults over the course of a year (40 per country, employing an innovative mobile phone technology), focus group discussions with study participants and structured assessments of health facilities (including formal and informal providers).

**Ethics and dissemination:**

Ethical approval has been granted by the Observational Research Ethics Committee at the London School of Hygiene and Tropical Medicine and the Research Ethics Boards at the Universiti Putra Malaysia and the University of the Philippines Manila. The project team will disseminate findings and engage with a wide range of stakeholders to promote uptake and impact. Alongside publications in high-impact journals, dissemination activities include a comprehensive stakeholder analysis, engagement with traditional and social media and ‘digital stories’ coproduced with research participants.

**Strengths and limitations of this study**

- Our prospective cohort design study benefits from a solid evidence base consisting of two systematic reviews.
- A key strength of the proposed study is its mixed-method design including innovative mobile phone technology to enhance examination of patient pathways.
- We may encounter limited access to facilities, as facility staff may be reluctant to participate or fully disclose information due to regulatory or commercial reasons.
- Participant attrition between the interview and follow-up interview and household survey phases may constitute a limitation to findings inferred from follow-up data.

## Background

Control of hypertension is an essential part of any strategy to reduce the burden of cardiovascular diseases (CVDs) worldwide.^1 2^ Malaysia and the Philippines are countries where hypertension control is poor (table 1), and many other low-income and middle-income countries (LMICs) face similar challenges.^3 4^ The poorest individuals in the lowest wealth quintile are especially disadvantaged,^4–7^ with profound implications for their health and economic well-being.^8^ As two recent systematic reviews showed, health systems and services barriers to effective management exist in countries at all levels of development but can be overcome.^9 10^ However, a comprehensive approach will also take account of patients’ sociocultural contexts, exploring how they interact with health services to facilitate or hinder hypertension control.

**Table 1:** Treatment gap in hypertension among adults aged 35–70

Country	Hypertension prevalence	% aware hypertensives	% of treated hypertensives	% of controlled hypertensives	% treated in wealthiest quintile	% treated in poorest quintile
Canada (comparator)	37.5	55.2	54.0	24.8	51.8	55.6
Malaysia	46.6	48.1	41.2	12.5	41.5	36.6
Philippines	51.2	54.5	46.1	13.5	64.5	34.1

Source: PURE Study.^4^

The core elements of hypertension management are well established, set out in the World Heart Federation Hypertension Roadmap and other guidelines for low-resource settings.^8 11^ However, their specific application in each country must take account of the lived experiences of those with hypertension and frontline healthcare providers, exploring understanding of the condition and treatment, actual sociocultural barriers to obtaining and adhering to treatment and how barriers can be overcome. Our previous research in Malaysia and Colombia demonstrated the importance of local context.^12 13^ Thus, the first objective of the Responsive and Equitable Health Systems— Partnership on Non-Communicable Diseases (RESPOND) project is to produce robust evidence on the barriers to effective hypertension management (ie, at diagnosis, treatment initiation and adherence and ultimately control) faced by poor households in Malaysia and the Philippines. This will be achieved through quantitative and qualitative observational methods in a longitudinal study design.

The experience of living with and managing hypertension is a dynamic process shaped by health services and people’s social contexts and life circumstances.^13–16^ Non-intrusive methods are needed to capture patients’ perspectives of the changing complexities of seeking care over time. Thus, our second objective is to evaluate opportunities offered by mobile phone technology to study the lived experience of hypertension and barriers people face in handling hypertension. Specifically, we will develop an innovative qualitative data collection method involving the use of real-time ‘digital diaries’ in both countries.

## Methods and study design

This is a prospective cohort design combining three quantitative and three qualitative elements to explore several research questions: How are patients with hypertension diagnosed? What pathways do patients follow through the key stages of care? What care is sought and received, and what are the economic and social costs? How does this differ by patient characteristic, and why? What barriers impede continuing access to care and medication? How does context affect experiences of care? How feasible and acceptable are ‘digital diaries’ for collecting longitudinal qualitative data on lived experiences with chronic illness?

**Figure 1:**
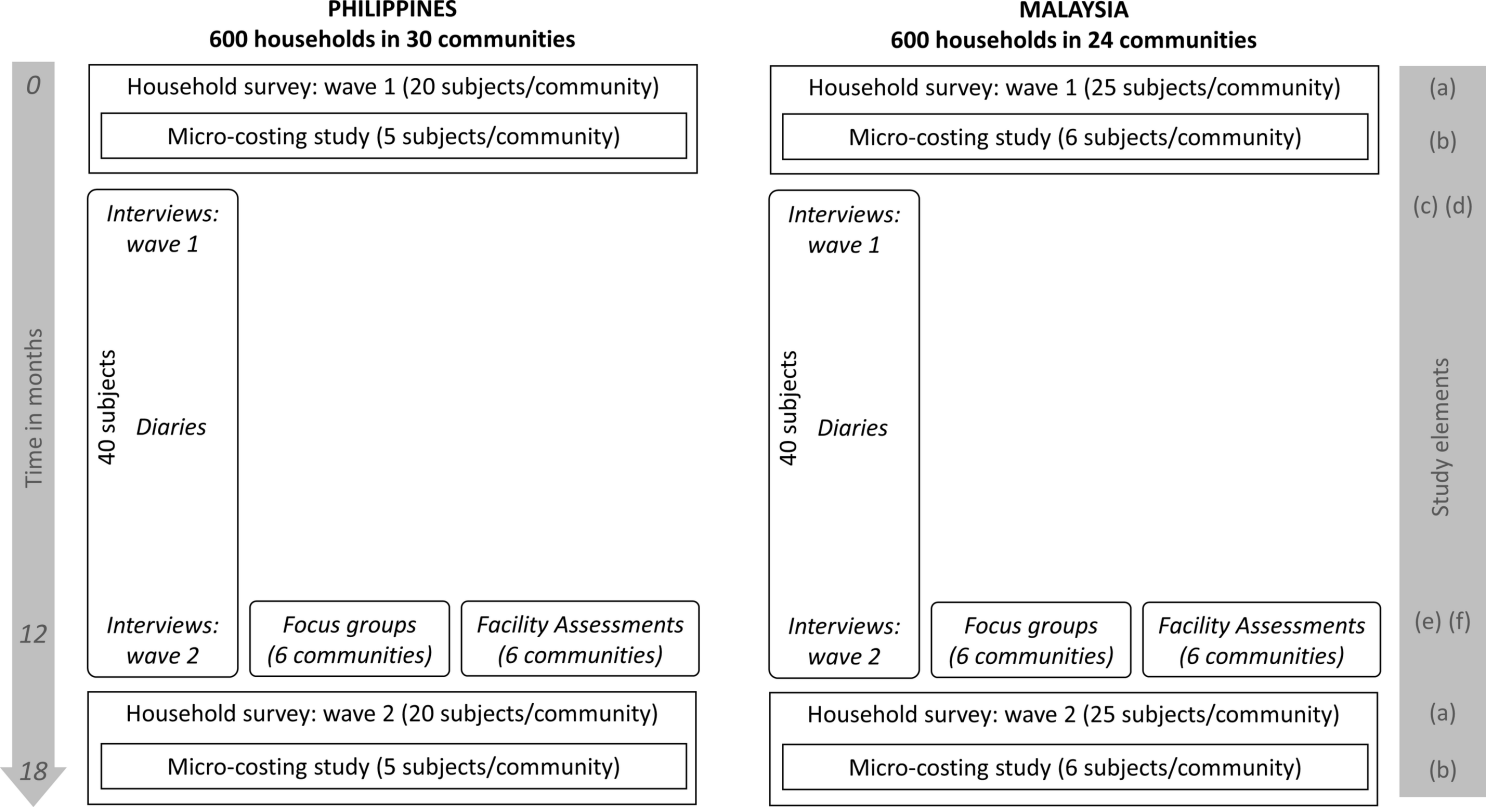
Study elements in Malaysia and the Philippines over 18 months.

In each country, the quantitative elements include: (A) 600 initial and follow-up household surveys (12–18 months apart) of patients with hypertension residing in 24–30 low-income rural and urban communities, (B) an embedded microcosting study of 5 hypertensive participants in each community and (C) approximately 200 structured facility assessments of hypertension care providers. The qualitative components are: (D) 40 initial and follow-up in-depth semistructured interviews among a subsample of survey participants with (E) up to 40 digital diaries recorded by interviewees between interviews and (F) 12 focus group discussions (FGDs) with hypertensive adults in 6 different communities. These elements are illustrated in figure 1.

### Initial and follow-up household surveys

In each country, 24–30 communities (*mukim* in Malaysia and *barangays* in the Philippines) will be selected, divided between urban and rural areas. In Malaysia, communities are in four peninsular states (Selangor, Kelantan, Perak and Johor). In the Philippines, 7 urban communities are in the City of Valenzuela in Metro Manila and 8 urban and 15 rural communities in Quezon province. While these have been purposefully selected to facilitate access by researchers, we will randomly select adults living within poor households (low-income households qualifying for government subsides under the BR1M programme in Malaysia^17^ and the 4P programme in the Philippines^18^) to obtain a statistically representative sample.

Within selected households, eligible individuals are: (1) aged 35–70 years at screening, (2) living within households expected to remain at the current address/location for at least 18 months and (3) either a self-reported history of hypertension or identified as hypertensive during screening. Individuals with a self-reported history of either cancer and/or HIV will be excluded, as their healthcare use is likely to be atypical.

After informed consent is taken, recruited participants’ blood pressure measurement will be taken using a digital sphygmomanometer following a standardised procedure. Hypertension is defined as blood pressure greater than or equal to 140/90 mm Hg or a self-reported history of hypertension.^19^ Among all hypertensive members in eligible households, one will be invited to participate using a probability based method, such as the KISH, age-order or full enumeration methods.^20^

A questionnaire consisting of validated instruments, including the Demographic & Health Surveys,^21^ WHO STEPS,^22^ World Values Survey^23^ and the Living Conditions, Lifestyle and Health Survey,^24^ will be administered to participants within their homes, collecting information on housing characteristics, including structure, amenities and household assets. A validated household asset-based wealth score, allowing within-country and cross-country comparisons, will be calculated.^25 26^ Detailed information from individuals and households will be collected, including age, gender, marital status, ethnicity, education, literacy, occupation; hypertension-related care experiences in the past 12 months (including service utilisation, settings (formal and informal practitioners, including criteria for choosing), treatment and adherence, transportation to care, knowledge of hypertension, attitudes to hypertension and its treatment); personal and environmental tobacco exposure; socioeconomic factors including employment income, education and psychosocial factors (social capital, attitudes and beliefs) and modifiable CVD risk factors for the ‘non-laboratory’ INTERHEART risk score.^27^ Data on healthcare pathways will be subdivided into discrete stages along the journey, collecting details of experiences at each, including duration, location, processes undertaken, treatment provided, costs and self-reported reasons for seeking care.^28^ A second wave of interviews, using an adapted version of the first instrument, will be conducted on the same participants after 12 months.

Sample size calculation took account of many possible analyses. For example, the ability to detect an urban-rural difference in treatment of hypertension in a middle-income country with α=0.05 and power of 0.8 (two-tailed), which one recent study suggested was as large as 13 percentage points (42% urban, 28% rural),^3^ would require a sample of 364 individuals with hypertension.

### Microcosting study

The microcosting study assesses the economic burden of hypertension on poor households. All who consent to participate in the household survey and are aware of their hypertension at baseline will be eligible to participate. Five in each community will be selected randomly to provide microcosting data. They will be asked additional questions related to household income and expenditure, health expenditure related to hypertension management, health financing (including insurance coverage) and coping strategies (labour substitution for ill person and/or caretaker, use of savings, changing consumption patterns, sale of assets, borrowing, other strategies). If participants cannot provide the requested information (eg, because they are not involved in the management of household finances), they will be asked to consult a knowledgeable household member. The same information will be collected at baseline and follow-up.

### Facilities assessment

Health system assessments will be undertaken contemporaneously with the second round of household surveys. These will provide ‘focused snapshots’ of the hypertension care available to communities. Facilities included are those where patients receive care for hypertension within the six communities in both countries where FGDs will be conducted (see below). They will be identified according to participant-led utility of healthcare providers, encompassing different levels of public, private, not-for-profit hospitals and clinics, complementary care providers, traditional and alternative healers, community health workers, outreach programmes/missions, retail pharmacies, drug stores or dispensaries, general retailers and/or mobile vendors. Within each of the 6 communities in both countries, a minimum of 2–3 providers of each type within the community will be included, generating up to 200 assessments across a very diverse range of providers.

Trained data collectors will use a structured facility questionnaire informed by the Service Availability and Readiness Assessments (SARA) developed by the WHO^29^ to characterise care in selected facilities, including infrastructure and work conditions; human resources; equipment, medicines and supplies; written information and educational materials.^30^ Questionnaires will also examine providers’ practices, perspectives and experiences and provide space for data collectors to provide free text information on their impressions and comments on the respondent, facility and any other observations.

### In-depth qualitative interviews

Initial and follow-up in-depth qualitative interviews will be conducted with a total of 80 participants (40 each in Malaysia and the Philippines). These will be purposively sampled from the household survey participants. The local teams will seek to recruit a balanced sample by sex, age, stage of treatment process, rural-urban locality and health complexity. Each participant will be interviewed shortly after completion of the household survey and again after 12–18 months have passed. Interviews will be conducted by the local research team and will be audio-recorded and transcribed verbatim for analysis. Information about hypertension services will be provided to all participants after the interview.

The interview will be conversational and explore participants’ views on hypertension, including treatment and care, as well as their own personal experiences of living with the condition and of accessing and using various types of healthcare services, including traditional and complementary medicine. We will attempt to understand people’s conception of hypertension (including its diagnosis and treatment) and lived healthcare experiences and trajectories in relation to their wider sociocultural and environmental contexts (eg, work, community and family relationships). Follow-up interviews will explore changes over time and issues raised in the first interview and during digital diary recording (see below). To address our secondary methodological objective, we will add additional questions for those who have completed digital diaries in order to explore their experiences of using the mobile-phone diary technology and to assess the overall feasibility of this qualitative data collection method.

### Digital diaries

All interview participants will be invited to complete optional digital diaries (via mobile phones) between the initial and follow-up interviews, yielding a maximum of 80 digital diaries from both countries. Those who consent but do not have a mobile phone will be provided with one (along with mobile phone credit) from the study team to allow the recording of their digital diary. Those who already possess mobile phones will only receive mobile phone credit. The participants will be trained on how to submit diary entries, provided with a guidance leaflet to keep after the training and continually engaged for in-depth information on patient experience.

Diary participants will be encouraged to record their experiences of living with hypertension, the barriers to treatment and control of hypertension they and their families face and, crucially, their view of feasible solutions. Participants will be able to submit audio (spoken), written (text messages) and visual (photo, video) material via mobile phones. Study team members will explain to participants that diary recording is totally voluntary and that they can record as much as they prefer. They will also discuss with them whether they would be happy to receive probes via text message from study members in some occasions. Through such probing, respondents can reflect on and reappraise their own experiences with hypertension and use of services and thus improve the depth of the data. If participants do not submit any entries in the first 2 months of enrolling into the study, they will be replaced with another participant.^31^

### Focus group discussions

Twelve FGDs with study participants will be held after the follow-up interviews have been completed, comprising six each from the Philippines and Malaysia. We aim for between 7 and 10 participants per FGD, yielding a total FGD sample between 84 and 120. At least one FGD will be conducted exclusively with participants who completed digital diaries. The study team will strive to match individuals with other FGD participants by age group and sex.

The focus groups offer an opportunity to access shared and conflicting aspects of the participant’s health beliefs and experiences and social processes that shape individual decision-making about treatment, accessing services and so on. The discussion will focus on trajectories of care and participants’ own prioritisation of issues and solutions.

### Patient and public involvement

The development of our research questions and study design was based on the premise that effective management of hypertension requires patient-centred health systems that respond to patient needs, health seeking behaviour and preferences, which often vary among and within countries. We have engaged with stakeholders and community representatives, including patient groups, during the project inception phase to identify major challenges faced by the poor and hard-to-reach communities, and to understand local culture and how it may affect the study. We will maintain this engagement throughout field work and analysis to ensure that we can respond to emerging changes in the field and allow adaptation in real time. In line with our patient-centred approach, several key outputs will be coproduced jointly between communities and researchers. Some of these outputs were designed explicitly to engage the public through digital stories, policy briefings, webcasts and interactive panels.

## Analysis

### Quantitative components

To describe pathways of care, we will draw on methods used previously to examine the unique sequences of care-seeking behaviours taken by hypertensive individuals to manage their condition.^28^ The resulting data will be used to analyse the determinants of hypertension detection, treatment and control using appropriate regression models. We will also use multilevel models that nest the experience of the individual within their community and health systems contexts, to obtain community-adjusted estimates.

The household economic burden of hypertension will be assessed by summing all direct and indirect costs incurred by households related to hypertension over the preceding 12 months. Indirect costs will be estimated using the human capital model,^32^ accounting for lost productivity due to morbidity and premature mortality. Morbidity costs are defined as lost income due to disability and inability to work as a result of hypertension and/or its consequences, calculated by multiplying total of days off work by a context-appropriate valuation of average gross daily earnings. Mortality costs are defined as lost income attributable to hypertension-related premature mortality, multiplying years of life lost by a context-appropriate valuation of annual net income. Years of life lost will be calculated using the Global Burden of Disease methods.^33^ Catastrophic health payment will be defined as occurring when health expenditures exceed 40% of a household’s non-food expenditures or capacity to pay for services, in the 12 months prior to the interview; a range of thresholds will be used for sensitivity analyses.

### Qualitative components

Analysis of qualitative data generated via interviews, digital diaries and FGDs will be conducted by local researchers with support by the London team. The analysis will focus on how participants (and carers) make sense of hypertension and the experience of living with and managing the condition and accessing care. It will also examine the temporal ordering of events in care seeking and management of hypertension, to understand links between actions and consequences over time and across contexts. A combined deductive and inductive approach will guide the analysis,^34^ with the process reviewed and refined iteratively. A coding framework will be developed using Nvivo 11 qualitative analysis software, followed by an analytical process of identifying, comparing and contrasting increasingly abstract themes, within and across countries.

### Data management

All digital data will be anonymised and transferred to secure servers. The data will be accessible to project members in the UK, Malaysia and the Philippines via LSHTM’s secure/password protected web portal. User authentication and encryption will be applied throughout. Laptops and data sticks used in data collection will be encrypted, and institutional networks are protected using user authentication. Any printed material used during transcription will be stored in a locked cabinet and destroyed when no longer needed.

### Dissemination

Our knowledge dissemination strategy includes publication of academic articles in high-impact journals, engagement with key stakeholders informed by a stakeholder analysis and contributions to traditional and social media.

## Discussion

The RESPOND project is particularly appropriate to assess the health needs of poor populations in Malaysia and the Philippines. The economic and treatment burden of hypertension is likely to disproportionately affect poor populations who suffer multiple disadvantage and substantial disease burden, both infectious and non-communicable. Means to address this issue, and the growing non-communicable disease (NCD) burden more broadly, is now an important policy priority. Evidence from the RESPOND study will promote essential insights to design and implement contextually appropriate initiatives.

While many of the findings from the RESPOND project will be specific to the two countries, some will be more widely applicable. The project will also inform the development of methods that can be used in other LMICs at a similar stage of the epidemiological transition, with relatively under-resourced health systems where patient-centred approaches focused on the needs of the poor can be further enhanced. This is facilitated by our framing of the research questions and design within the global literature on NCD, ensuring possibilities for comparisons with other settings, supporting exposure of the researchers involved to international debates and engagement with dissemination activities at global and national levels.

## Supplementary Material

Reviewer comments

Author's manuscript
