# Supplementary material for: Responsive and Equitable Health Systems—Partnership on Non-Communicable Diseases (RESPOND) study: a mixed-methods, longitudinal, observational study on treatment seeking for hypertension in Malaysia and the Philippines
Source: BMJ Open. 2018 Jul 30;8(7):e024000. doi: 10.1136/bmjopen-2018-024000 (PMC6067392; doi:10.1136/bmjopen-2018-024000)
Supplement: Author's manuscript [file bmjopen-2018-024000.draft_revisions.pdf]

BMJ Open is committed to open peer review. As part of this commitment we make the peer review history of every article we publish publicly available.

When an article is published we post the peer reviewers' comments and the authors' responses online. We also post the versions of the paper that were used during peer review. These are the versions that the peer review comments apply to.

The versions of the paper that follow are the versions that were submitted during the peer review process. They are not the versions of record or the final published versions. They should not be cited or distributed as the published version of this manuscript.

BMJ Open is an open access journal and the full, final, typeset and author-corrected version of record of the manuscript is available on our site with no access controls, subscription charges or pay-per-view fees (http://bmjopen.bmj.com).

If you have any questions on BMJ Open's open peer review process please email info.bmjopen@bmj.com

BMJ Open

The Responsive and Equitable Health Systems – Partnership on Non-Communicable Diseases (RESPOND) study: A mixed-methods, longitudinal, observational study on treatment seeking for hypertension in Malaysia and the Philippines

Journal:	*BMJ Open*
Manuscript ID	bmjopen-2018-024000
Article Type:	Protocol
Date Submitted by the Author:	10-May-2018

Complete List of Authors:	Palafox, B; The London School of Hygiene and Tropical Medicine, Centre for Global Chronic Conditions<br>Seguin, Maureen; The London School of Hygiene and Tropical Medicine, The Centre for Global Chronic Conditions<br>McKee, Martin; The London School of Hygiene and Tropical Medicine, The Centre for Global Chronic Conditions<br>Dans, Antonio; University of the Philippines, College of Medicine<br>Yusoff, Khalid; UCSI University, Faculty of Medicine and Health Sciences; University Teknologi MARA, Centre for Translational Research & Epidemiology, Faculty of Medicine<br>Candari, Christine; University of the Philippines, College of Medicine<br>Idris, Khairuddin; University Putra Malaysia, Institute for Social Science Studies<br>Ismail, Johan; University Teknologi MARA, Centre for Translational Research & Epidemiology, Faculty of Medicine<br>Krauss, Steven; University Putra Malaysia, Institute for Social Science Studies<br>Lasco, Gideon; University of the Philippines Diliman, Department of Anthropology<br>Majid, Fadhlina; University Teknologi MARA, Centre for Translational Research & Epidemiology, Faculty of Medicine<br>Palileo-Villanueva, Lia; University of the Philippines, College of Medicine<br>Razak, Azlina; UCSI University, Faculty of Medicine and Health Sciences<br>Renedo, Alicia; The London School of Hygiene and Tropical Medicine, Social and Environmental Health Resarch<br>Balabanova, Dina; The London School of Hygiene and Tropical Medicine, The Centre for Global Chronic Conditions

Keywords:	Hypertension < CARDIOLOGY, Health systems, Cardiovascular disease, Malaysia, Philippines

SCHOLARONE™ Manuscripts

For peer review only

Title: The Responsive and Equitable Health Systems – Partnership on Non-Communicable Diseases (RESPOND) study: A mixed-methods, longitudinal, observational study on treatment seeking for hypertension in Malaysia and the Philippines

Corresponding author:
Palafox, Benjamin, London School of Hygiene & Tropical Medicine, 15-17 Tavistock Place, Kings Cross, London WC1H 9SH, benjamin.Palafox@lshtm.ac.uk, 02079588337

Full names, departments, institutions, city and country of all co-authors:
Palafox, Benjamin*, Centre for Global Chronic Conditions, London School of Hygiene and Tropical Medicine, London, UK

Seguin, Maureen L*, Centre for Global Chronic Conditions, London School of Hygiene and Tropical Medicine, London, UK

McKee, Martin, Centre for Global Chronic Conditions, London School of Hygiene and Tropical Medicine, London, UK

Dans, Antonio L, College of Medicine, University of the Philippines Manila, Manila, PH

Yusoff, Khalid, Faculty of Medicine and Health Sciences, UCSI University, Kuala Lumpur, MY and Centre for Translational Research & Epidemiology, Faculty of Medicine, University Teknologi MARA, Shah Alam, MY

Candari, Christine J, College of Medicine, University of the Philippines Manila, Manila, PH

Idris, Khairuddin, Institute for Social Science Studies, University Putra Malaysia, Serdang, MY

Ismail, Johan Rizwal, Centre for Translational Research & Epidemiology, Faculty of Medicine, University Teknologi MARA, Shah Alam, MY

Krauss, Steven Eric, Institute for Social Science Studies, University Putra Malaysia, Serdang, MY

Lasco, Gideon, Department of Anthropology, University of the Philippines Diliman, Quezon City, PH
Majid, Fadhlina A, Centre for Translational Research & Epidemiology, Faculty of Medicine, University Teknologi MARA, Shah Alam, MY

Palileo-Villanueva, Lia M, College of Medicine, University of the Philippines Manila, Manila, PH

Razak, Azlina A, Faculty of Medicine and Health Sciences, UCSI University, Kuala Lumpur, MY

Renedo, Alicia, Department of Social and Environmental Health Research, London School of Hygiene and Tropical Medicine, London, UK

For peer review only - http://bmjopen.bmj.com/site/about/guidelines.xhtml

Balabanova, Dina, Centre for Global Chronic Conditions, London School of Hygiene and Tropical Medicine, London, UK

*Benjamin Palafox and Maureen Seguin contributed equally to this paper

Keywords:
Hypertension, Health systems, Cardiovascular disease, Malaysia, Philippines

Word count excluding title page, abstract, references, figures and tables: 2952

Reference count: 34

For peer review only

ABBREVIATIONS

CVD cardiovascular disease, FGD focus group discussion, LMIC low- and middle-income country, RESPOND Responsive and Equitable Health Systems–Partnership on Non-Communicable Diseases, SARA Service Availability and Readiness Assessments

ABSTRACT

Introduction: Hypertension is a leading contributor to the global burden of disease. While safe and effective treatment exists, blood pressure control is poor in many countries, often reflecting barriers at the levels of health systems and services, as well as at the broader level of patients' socio-cultural contexts. This study examines how these interact to facilitate or hinder hypertension control, taking into account characteristics of service provision components and social contexts.

Methods and Analysis: The study, set in Malaysia and the Philippines, builds on two systematic reviews of barriers to effective hypertension management. People with hypertension (pre-existing and newly diagnosed) will be identified in poor households in 24-30 communities per country. Quantitative and qualitative methods will be used to examine their experiences of and pathways into seeking and obtaining care. These include two waves of household surveys of 20-25 participants per community 12-18 months apart, micro-costing exercises to assess the cost of illness (including costs due to health seeking activities and inability to work [5 per community]), preliminary and follow-up in-depth interviews and digital diaries with hypertensive adults over the course of a year (40 per country, employing an innovative mobile phone technology), focus group discussions with study participants, and structured assessments of health facilities (including formal and informal providers).

Ethics and Dissemination: Ethical approval has been granted by the Observational Research Ethics Committee at the London School of Hygiene and Tropical Medicine, and the Research Ethics Boards at the Universiti Putra Malaysia and the University of the Philippines Manila. The project team will disseminate findings and engage with a wide range of stakeholders to promote uptake and impact. Alongside publications in high-impact journals, dissemination activities include a comprehensive stakeholder analysis, engagement with traditional and social media and 'digital stories' co-produced with research participants.

For peer review only - http://bmjopen.bmj.com/site/about/guidelines.xhtml

STRENGTHS AND LIMITATIONS

- Our prospective cohort design study benefits from a solid evidence base consisting of two systematic reviews.
- A key strength of the proposed study is its mixed-method design including innovative mobile phone technology to enhance examination of patient pathways.
- We may encounter limited access to facilities, as facility staff may be reluctant to participate or fully disclose information due to for regulatory or commercial reasons.
- Participant attrition between the interview and follow-up interview and household survey phases may constitute a limitation to findings inferred from follow-up data.

BACKGROUND

Control of hypertension is an essential part of any strategy to reduce the burden of cardiovascular diseases (CVDs) worldwide.[1 2] Malaysia and the Philippines are countries where hypertension control is poor (Table 1), and many other low- and middle-income countries (LMICs) face similar challenges.[3 4] The poorest individuals in the lowest wealth quintile are especially disadvantaged, [4-7] with profound implications for their health and economic wellbeing.[8] As two recent systematic reviews showed, health systems and services barriers to effective management exist in countries at all levels of development but can be overcome.[9 10] However, a comprehensive approach will also take account of patients' socio-cultural contexts, exploring how they interact with health services to facilitate or hinder hypertension control.

Table 1: Treatment gap in hypertension among adults aged 35-70

Country	*Hypertension prevalence*	*% aware hypertensives*	*% of treated hypertensives*	*% of controlled hypertensives*	*% treated in wealthiest quintile*	*% treated in poorest quintile*
Canada (comparator)	*37.5*	*55.2*	*54.0*	*24.8*	*51.8*	*55.6*
Malaysia	*46.6*	*48.1*	*41.2*	*12.5*	*41.5*	*36.6*
Philippines	*51.2*	*54.5*	*46.1*	*13.5*	*64.5*	*34.1*

Source: PURE Study[4]

The core elements of hypertension management are well established, set out in the World Heart Federation Hypertension Roadmap and other guidelines for low-resource settings.[8 11] However, their specific application in each country must take account of the lived experiences of those with hypertension and frontline healthcare providers, exploring understanding of the condition and treatment, actual socio-cultural barriers to obtaining and adhering to treatment, and how barriers can be overcome. Our previous research in Malaysia and Colombia demonstrated the importance of local context.[12 13] Thus, the first objective of the Responsive and Equitable Health Systems – Partnership on Non-Communicable Diseases (RESPOND) project is to produce robust evidence on the barriers to effective hypertension management (i.e. at diagnosis, treatment initiation and adherence, and ultimately control) faced by poor households in Malaysia and the Philippines. This will be achieved through quantitative and qualitative observational methods in a longitudinal study design.

The experience of living with and managing hypertension is a dynamic process shaped by health services and people's social contexts and life circumstances.[13-16] Non-intrusive methods are needed to capture patients' perspectives of the changing complexities of seeking care over time. Thus, our second objective is to evaluate opportunities offered by mobile phone technology to study the lived

For peer review only - http://bmjopen.bmj.com/site/about/guidelines.xhtml

experience of hypertension and barriers people face in handling hypertension. Specifically, we will develop an innovative qualitative data collection method involving the use of real-time 'digital diaries' in both countries.

METHODS AND STUDY DESIGN

This is a prospective cohort design combining 3 quantitative and 3 qualitative elements to explore several research questions: How are patients with hypertension diagnosed? What pathways do patients follow through the key stages of care? What care is sought and received, and what are the economic and social costs? How does this differ by patient characteristic, and why? What barriers impede continuing access to care and medication? How does context affect experiences of care? How feasible and acceptable are 'digital diaries' for collecting longitudinal qualitative data on lived experiences with chronic illness?

In each country, the quantitative elements include: (a) 600 initial and follow-up household surveys (12-18 months apart) of hypertensive patients residing in 24-30 low-income rural and urban communities, (b) an embedded micro-costing study of 5 hypertensive participants in each community, and (c) approximately 200 structured facility assessments of hypertension care providers. The qualitative components are: (d) 40 initial and follow-up in-depth semi-structured interviews among a sub-sample of survey participants with (e) up to 40 digital diaries recorded by interviewees between interviews, and (f) 12 focus group discussions with hypertensive adults in 6 different communities. These elements are illustrated in Figure 1.

Initial and follow-up household surveys

In each country, 24-30 communities (*mukim* in Malaysia and *barangays* in the Philippines) will be selected, divided between urban and rural areas. In Malaysia, communities are in 4 peninsular states (Selangor, Kelantan, Perak and Johor). In the Philippines, 7 urban communities are in the City of Valenzuela in Metro Manila, and 8 urban and 15 rural communities in Quezon province. While these have been purposefully selected to facilitate access by researchers, we will randomly select adults living within poor households (low-income households qualifying for government subsides under the BR1M programme in Malaysia,[17] and the 4P programme in the Philippines[18]) to obtain a statistically representative sample.

Within selected households, eligible individuals are: a) aged 35-70 years at screening, b) living within households expected to remain at the current address/location for at least 18 months, and c) either a self-reported history of hypertension or identified as hypertensive during screening. Individuals with a self-reported history of either cancer and/or HIV will be excluded, as their health care use is likely to be atypical.

After informed consent is taken, recruited participants' blood pressure measurement will be taken using a digital sphygmomanometer following a standardised procedure. Hypertension is defined as blood pressure greater than or equal to 140/90 mm Hg or a self-reported history of hypertension.[19] Among all hypertensive members in eligible households, one will be invited to participate using a probability based method, such as the KISH, age-order or full enumeration methods.[20]

A questionnaire consisting of validated instruments, including the Demographic & Health Surveys,[21] WHO STEPS,[22] World Values Survey,[23] and the Living Conditions, Lifestyle and Health Survey,[24] will be administered to participants within their homes, collecting information on housing characteristics, including structure, amenities and household assets. A validated household asset-based wealth score, allowing within- and cross-country comparisons, will be calculated.[25 26] Detailed information from individuals and households will be collected, including age, gender, marital status, ethnicity,

For peer review only - http://bmjopen.bmj.com/site/about/guidelines.xhtml

education, literacy, occupation; hypertension-related care experiences in the past 12 months (including service utilisation, settings [formal and informal practitioners, including criteria for choosing], treatment and adherence, transportation to care, knowledge of hypertension, attitudes to hypertension and its treatment); personal and environmental tobacco exposure; socioeconomic factors including employment income, education, and psychosocial factors (social capital, attitudes and beliefs), and modifiable CVD risk factors for the 'non-laboratory' INTERHEART risk score.[27] Data on health care pathways will be subdivided into discrete stages along the journey, collecting details of experiences at each, including duration, location, processes undertaken, treatment provided, costs, and self-reported reasons for seeking care.[28] A second wave of interviews, using an adapted version of the first instrument, will be conducted on the same participants after 12 months.

Sample size calculation took account of many possible analyses. For example, the ability to detect an urban-rural difference in treatment of hypertension in a middle-income country with $\alpha=0.05$ and power of 0.8 (two-tailed), which one recent study suggested was as large as 13 percentage points (42% urban, 28% rural),[3] would require a sample of 364 individuals with hypertension.

Micro-costing study

The micro-costing study assesses the economic burden of hypertension on poor households. All who consent to participate in the household survey and are aware of their hypertension at baseline will be eligible to participate. Five in each community will be selected randomly to provide micro-costing data. They will be asked additional questions related to household income and expenditure, health expenditure related to hypertension management, health financing (including insurance coverage) and coping strategies (labour substitution for ill person and/or caretaker, use of savings, changing consumption patterns, sale of assets, borrowing, other strategies). If participants cannot provide the requested information (e.g. because they are not involved in the management of household finances), they will be asked to consult a knowledgeable household member. The same information will be collected at baseline and follow-up.

Facilities assessment

Health system assessments will be undertaken contemporaneously with the second round of household surveys. These will provide 'focused snapshots' of the hypertension care available to communities. Facilities included are those where patients receive care for hypertension within the 6 communities in both countries where focus group discussions will be conducted (see below). They will be identified according to participant-led utility of health care providers, encompassing different levels of public, private, not-for-profit hospitals and clinics, complementary care providers, traditional and alternative healers, community health workers, outreach programmes/missions, retail pharmacies, drug stores or dispensaries, general retailers, and/or mobile vendors. Within each of the 6 communities in both countries, a minimum of 2-3 providers of each type within the community will be included, generating up to 200 assessments across a very diverse range of providers.

Trained data collectors will use a structured facility questionnaire informed by the Service Availability and Readiness Assessments (SARA) developed by the World Health Organisation[29] to characterise care in selected facilities, including infrastructure and work conditions; human resources; equipment, medicines and supplies; written information and educational materials.[30] Questionnaires will also examine providers' practices, perspectives and experiences, and provide space for data collectors to provide free text information on their impressions and comments on the respondent, facility and any other observations.

In-depth qualitative interviews

For peer review only - http://bmjopen.bmj.com/site/about/guidelines.xhtml

Initial and follow-up in-depth qualitative interviews will be conducted with a total of 80 participants (40 each in Malaysia and the Philippines). These will be purposively sampled from the household survey participants. The local teams will seek to recruit a balanced sample by sex, age, stage of treatment process, rural-urban locality, and health complexity. Each participant will be interviewed shortly after completion of the household survey and again after 12-18 months have passed. Interviews will be conducted by the local research team and will be audio-recorded and transcribed verbatim for analysis. Information about hypertension services will be provided to all participants after the interview.

The interview will be conversational and explore participants' views on hypertension, including treatment and care, as well as their own personal experiences of living with the condition and of accessing and using various types of healthcare services, including traditional and alternative medicine. We will attempt to understand people's conception of hypertension (including its diagnosis and treatment) and lived healthcare experiences and trajectories in relation to their wider socio-cultural and environmental contexts (e.g. work, community, and family relationships). Follow-up interviews will explore changes over time and issues raised in the first interview and during digital diary recording (see below). To address our secondary methodological objective, we will add additional questions for those who have completed digital diaries in order to explore their experiences of using the mobile-phone diary technology and to assess the overall feasibility of this qualitative data collection method.

Digital diaries

All interview participants will be invited to complete optional digital diaries (via mobile phones) between the initial and follow-up interviews, yielding a maximum of 80 digital diaries from both countries. Those who consent but do not have a mobile phone will be provided with one (along with mobile phone credit) from the study team to allow the recording of their digital diary. Those who already possess mobile phones will only receive mobile phone credit. The participants will be trained on how to submit diary entries, provided with a guidance leaflet to keep after the training, and continually engaged for in-depth information on patient experience.

Diary participants will be encouraged to record their experiences of living with hypertension, the barriers to treatment and control of hypertension they and their families face and, crucially, their view of feasible solutions. Participants will be able to submit audio (spoken), written (text messages), and visual (photo, video) material via mobile phones. Study team members will explain to participants that diary recording is totally voluntary, and that they can record as much as they prefer. They will also discuss with them whether they would be happy to receive probes via text message from study members in some occasions. Through such probing, respondents can reflect upon and reappraise their own experiences with hypertension and use of services, and thus improve the depth of the data. If participants do not submit any entries in the first two months of enrolling into the study, they will be replaced with another participant.[31]

Focus group discussions

Twelve focus group discussions (FGDs) with study participants will be held after the follow-up interviews have been completed, comprising six each from the Philippines and Malaysia. We aim for between 7 and 10 participants per FGD, yielding a total FGD sample between 84 and 120. At least one FGD will be conducted exclusively with participants who completed digital diaries. The study team will strive to match individuals with other FGD participants by age group and sex.

The focus groups offer an opportunity to access shared and conflicting aspects of the participant's health beliefs and experiences and social processes that shape individual decision-making about

For peer review only - http://bmjopen.bmj.com/site/about/guidelines.xhtml

treatment, accessing services etc. The discussion will focus on trajectories of care and participants' own prioritisation of issues and solutions.

Patient and Public Involvement

The development of our research questions and study design was based on the premise that effective management of hypertension requires patient-centred health systems that respond to patient needs, health seeking behaviour and preferences, which often vary among and within countries. We have engaged with stakeholders and community representatives, including patient groups, during the project inception phase to identify major challenges faced by the poor and hard-to-reach communities, and to understand local culture and how it may affect the study. We will maintain this engagement throughout field work and analysis to ensure that we can respond to emerging changes in the field and allow adaptation in real time. In line with our patient-centred approach, several key outputs will be co-produced jointly between communities and researchers. Some of these outputs were designed explicitly to engage the public through digital stories, policy briefings, webcasts, and interactive panels.

ANALYSIS

Quantitative components

To describe pathways of care, we will draw on methods used previously to examine the unique sequences of care-seeking behaviours taken by hypertensive individuals to manage their condition.[28] The resulting data will be used to analyse the determinants of hypertension detection, treatment and control using appropriate regression models. We will also use multi-level models that nest the experience of the individual within their community and health systems contexts, to obtain community-adjusted estimates.

The household economic burden of hypertension will be assessed by summing all direct and indirect costs incurred by households related to hypertension over the preceding 12 months. Indirect costs will be estimated using the human capital model,[32] accounting for lost productivity due to morbidity and premature mortality. Morbidity costs are defined as lost income due to disability and inability to work as a result of hypertension and/or its consequences, calculated by multiplying total of days off work by a context-appropriate valuation of average gross daily earnings. Mortality costs are defined as lost income attributable to hypertension-related premature mortality, multiplying years of life lost by a context-appropriate valuation of annual net income. Years of life lost will be calculated using the Global Burden of Disease methods.[33] Catastrophic health payment will be defined as occurring when health expenditures exceed 40% of a household's non-food expenditures or capacity to pay for services, in the 12 months prior to the interview; a range of thresholds will be used for sensitivity analyses.

Qualitative components

Analysis of qualitative data generated via interviews, digital diaries, and FGDs will be conducted by local researchers with support by the London team. The analysis will focus on how participants (and carers) make sense of hypertension and the experience of living with and managing the condition and accessing care. It will also examine the temporal ordering of events in care seeking and management of hypertension, to understand links between actions and consequences over time and across contexts. A combined deductive and inductive approach will guide the analysis,[34] with the process reviewed and refined iteratively. A coding framework will be developed using Nvivo 11 qualitative analysis software, followed by an analytical process of identifying, comparing, and contrasting increasingly abstract themes, within and across countries.

Data management

For peer review only - http://bmjopen.bmj.com/site/about/guidelines.xhtml

All digital data will be anonymised and transferred to secure servers. The data will be accessible to project members in the UK, Malaysia, and the Philippines via LSHTM's secure/password protected web portal. User authentication and encryption will be applied throughout. Laptops and data sticks used in data collection will be encrypted, and institutional networks are protected using user authentication. Any printed material used during transcription will be stored in a locked cabinet and destroyed when no longer needed.

Ethics and dissemination

Ethical approval has been granted by the LSHTM Observational Research Ethics Committee and the Research Ethics Boards at the Universiti Putra Malaysia and the University of the Philippines Manila. Our knowledge dissemination strategy includes publication of academic articles in high-impact journals, engagement with key stakeholders informed by a stakeholder analysis, and contributions to traditional and social media.

DISCUSSION

The RESPOND project is particularly appropriate to assess the health needs of poor populations in Malaysia and the Philippines. The economic and treatment burden of hypertension is likely to disproportionately affect poor populations who suffer multiple disadvantage and substantial disease burden, both infectious and non-communicable. Means to address this issue, and the growing NCD burden more broadly, is now an important policy priority. Evidence from the RESPOND study will promote essential insights to design and implement contextually appropriate initiatives.

While many of the findings from the RESPOND project will be specific to the two countries, some will be more widely applicable. The project will also inform the development of methods that can be used in other low- and middle-income countries at a similar stage of the epidemiological transition, with relatively under-resourced health systems where patient-centred approaches focused on the needs of the poor can be further enhanced. This is facilitated by our framing of the research questions and design within the global literature on NCD, ensuring possibilities for comparisons with other settings, supporting exposure of the researchers involved to international debates and engagement with dissemination activities at global and national levels.

ACKNOWLEDGMENTS

The authors would like to thank the Wellcome Trust/Newton Fund-MRC Humanities & Social Science Collaborative Award scheme for providing funding for this research.

FUNDING

This study is supported by a grant from the Wellcome Trust/Newton Fund-MRC Humanities & Social Science Collaborative Award scheme (200346/Z/15/Z).

CONTRIBUTORS

BP, MM, DB, AAR, ALD and KY conceptualised the study. BP, MS, MM, ALD, KY, CC, KI, JRI, SEK, GL, FAM, LMPV, AAR, AR, and DB contributed to the development of study design. BP, MS, MM, ALD, KY, CC, KI, JRI, SEK, GL, FAM, LMPV, AAR, AR, and DB read, edited, and approved the final version. MS and BP contributed equally to the first draft of the manuscript.

COMPETING INTERESTS

For peer review only - http://bmjopen.bmj.com/site/about/guidelines.xhtml

For peer review only

ALD, KY, JRI, FAM and LMPV have been and/or are currently involved in clinical trials of antihypertensive medications that receive some funding from industry.

REFERENCES

1. GBD 2016 SDG Collaborators. Measuring progress and projecting attainment on the basis of past trends of the health-related Sustainable Development Goals in 188 countries: an analysis from the Global Burden of Disease Study 2016. *Lancet (London, England)* 2017;390(10100):1423-59. doi: 10.1016/s0140-6736(17)32336-x [published Online First: 2017/09/17]

2. GBD 2016 Risk Factors Collaborators. Global, regional, and national comparative risk assessment of 84 behavioural, environmental and occupational, and metabolic risks or clusters of risks, 1990-2016: a systematic analysis for the Global Burden of Disease Study 2016. *Lancet (London, England)* 2017;390(10100):1345-422. doi: 10.1016/s0140-6736(17)32366-8 [published Online First: 2017/09/19]

3. Chow CK, Teo KK, Rangarajan S, et al. Prevalence, awareness, treatment, and control of hypertension in rural and urban communities in high-, middle-, and low-income countries. *JAMA* 2013;310(9):959-68. doi: 10.1001/jama.2013.184182

4. Palafox B, McKee M, Balabanova D, et al. Wealth and cardiovascular health: a cross-sectional study of wealth-related inequalities in the awareness, treatment and control of hypertension in high-, middle- and low-income countries. *International Journal for Equity in Health* 2016;11(199) doi: 10.1186/s12939-016-0478-6

5. Lloyd-Sherlock P, Beard J, Minicuci N, et al. Hypertension among older adults in low- and middle-income countries: prevalence, awareness and control. *International Journal of Epidemiology* 2014 doi: 10.1093/ije/dyt215

6. Mendez MA, Cooper R, Wilks R, et al. Income, education, and blood pressure in adults in Jamaica, a middle-income developing country. *Int J Epidemiol* 2003;32(3):400-8.

7. Moser KA, Agrawal S, Davey Smith G, et al. Socio-Demographic Inequalities in the Prevalence, Diagnosis and Management of Hypertension in India: Analysis of Nationally-Representative Survey Data. *PLoS ONE* 2014;9(1):e86043. doi: 10.1371/journal.pone.0086043

8. Adler AJ, Prabhakaran D, Bovet P, et al. Reducing Cardiovascular Mortality Through Prevention and Management of Raised Blood Pressure: A World Health Federation Roadmap. *Global Heart* 2015;10(2):111-22. doi: 10.1016/j.gheart.2015.04.006

9. Khatib R, Schwalm J-D, Yusuf S, et al. Patient and Healthcare Provider Barriers to Hypertension Awareness, Treatment and Follow Up: A Systematic Review and Meta-Analysis of Qualitative and Quantitative Studies. *PLoS ONE* 2014;9(1):e84238. doi: 10.1371/journal.pone.0084238

10. Maimaris W, Paty J, Perel P, et al. The influence of health systems on hypertension awareness, treatment, and control: a systematic literature review. *PLoS medicine* 2013;10(7):e1001490. doi: 10.1371/journal.pmed.1001490

11. Modesti PA, Agostoni P, Agyemang C, et al. Cardiovascular risk assessment in low-resource settings: a consensus document of the European Society of Hypertension Working Group on Hypertension and Cardiovascular Risk in Low Resource Settings. *J Hypertens* 2014;32(5):951-60. doi: 10.1097/hjh.0000000000000125 [published Online First: 2014/03/01]

For peer review only - http://bmjopen.bmj.com/site/about/guidelines.xhtml

12. Risso-Gill I, Balabanova D, Majid F, et al. Understanding the modifiable health systems barriers to hypertension management in Malaysia: a multi-method health systems appraisal approach. *BMC Health Serv Res* 2015;15:254. doi: 10.1186/s12913-015-0916-y
13. Legido-Quigley H, Camacho Lopez PA, Balabanova D, et al. Patients' knowledge, attitudes, behaviour and health care experiences on the prevention, detection, management and control of hypertension in Colombia: a qualitative study. *PLoS One* 2015;10(4):e0122112. doi: 10.1371/journal.pone.0122112
14. Morgan M, Watkins CJ. Managing hypertension: beliefs and responses to medication among cultural groups. *Sociology of Health & Illness* 1988;10(4):561-78. doi: 10.1111/1467-9566.ep10837256
15. Marshall IJ, Wolfe CDA, McKevitt C. Lay perspectives on hypertension and drug adherence: systematic review of qualitative research. *BMJ : British Medical Journal* 2012;345 doi: 10.1136/bmj.e3953
16. Higginbottom GM. 'Pressure of life': ethnicity as a mediating factor in mid-life and older peoples' experience of high blood pressure. *Sociol Health Illn* 2006;28(5):583-610. doi: 10.1111/j.1467-9566.2006.00508.x [published Online First: 2006/08/17]
17. Kamaruddin R, Othman AA, Denan Z. Government Sincere Initiatives or Political Motives of 1Malaysia Peoples' Aid: Using Structural Equation Modeling. *Procedia - Social and Behavioral Sciences* 2013;105(Supplement C):715-22. doi: https://doi.org/10.1016/j.sbspro.2013.11.074
18. Republic of the Philippines. Pantawid Pamilyang Pilipino Program (4P) Manila: Government of the Philippines; 2017 [Available from: http://www.officialgazette.gov.ph/programs/conditional-cash-transfer/ accessed 10 November 2017.
19. Gee ME, Campbell N, Sarrafzadegan N, et al. Standards for the uniform reporting of hypertension in adults using population survey data: recommendations from the World Hypertension League Expert Committee. *Journal of clinical hypertension* 2014;16(11):773-81. doi: 10.1111/jch.12387
20. Gaziano C. Comparative Analysis of Within-Household Respondent Selection Techniques. *Public Opinion Quarterly* 2005;69(1):124-57. doi: 10.1093/poq/nfi006
21. USAID. Demographic and Health Surveys 2017 [Available from: https://dhsprogram.com/ accessed 10th October 2017.
22. World Health Organization. WHO STEPS surveillance manual: the WHO STEPwise approach to chronic disease risk factor surveillance. Geneva: WHO 2005.
23. Inglehart R, Puranen B, Pettersson T, et al. The World Values Survey. 2005
24. Balabanova D, Roberts B, Richardson E, et al. Health care reform in the former Soviet Union: beyond the transition. *Health services research* 2012;47(2):840-64. doi: 10.1111/j.1475-6773.2011.01323.x [published Online First: 2011/11/19]
25. Filmer D, Pritchett LH. Estimating wealth effects without expenditure data--or tears: an application to educational enrollments in states of India. *Demography* 2001;38(1):115-32. [published Online First: 2001/03/03]
26. Vyas S, Kumaranayake L. Constructing socio-economic status indices: how to use principal components analysis. *Health Policy Plan* 2006;21(6):459-68. doi: 10.1093/heapol/czl029
27. Yusuf S, Hawken S, Ounpuu S, et al. Effect of potentially modifiable risk factors associated with myocardial infarction in 52 countries (the INTERHEART study): case-

For peer review only - http://bmjopen.bmj.com/site/about/guidelines.xhtml

control study. *Lancet* 2004;364(9438):937-52. doi: 10.1016/s0140-6736(04)17018-9 [published Online First: 2004/09/15]
28. Haenssgen MJ, Ariana P. Healthcare access: A sequence-sensitive approach. *SSM - Population Health* 2017;3:37-47. doi: http://dx.doi.org/10.1016/j.ssmph.2016.11.008
29. Organization WH. Service availability and readiness assessment (SARA): an annual monitoring system for service delivery: reference manual. Geneva: WHO 2013.
30. O'Neill K, Takane M, Sheffel A, et al. Monitoring service delivery for universal health coverage: the Service Availability and Readiness Assessment. *Bulletin of the World Health Organization* 2013;91:923-31.
31. Patton MQ. Qualitative research and evaluation methods. 2 ed. London: Sage Publications 2002.
32. Pritchard C, Sculpher M. Productivity Costs: Principles and Practice in Economic Evaluation. London: Office of Health Economics 2000.
33. Murray CJL, Lopez AD. Measuring the Global Burden of Disease. *New England Journal of Medicine* 2013;369(5):448-57. doi: 10.1056/NEJMra1201534
34. Braun V, Clarke V. Using thematic analysis in psychology. *Qualitative Research in Psychology* 2006;3(2):77-101.

For peer review only

For peer review only - http://bmjopen.bmj.com/site/about/guidelines.xhtml

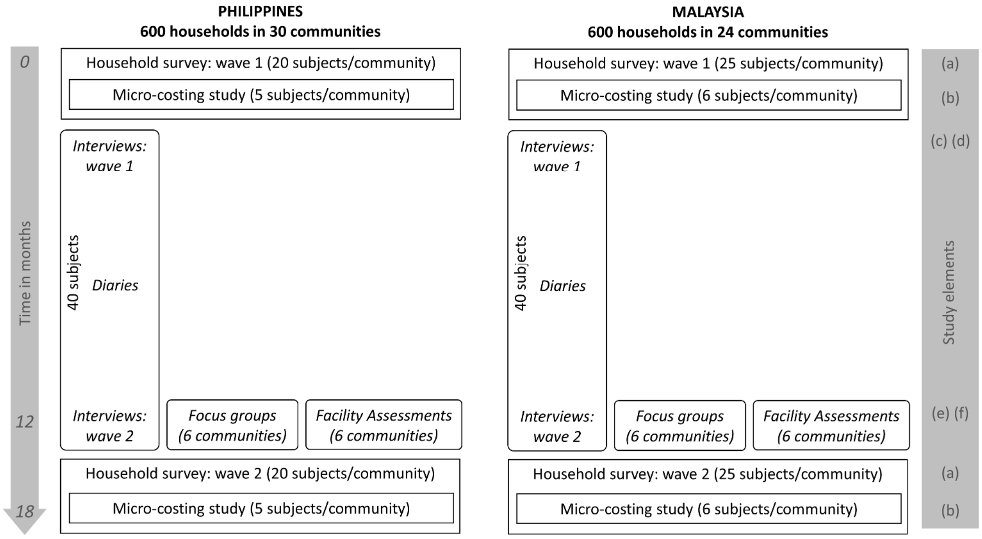

Study elements in Malaysia and the Philippines over 18 months

338x190mm (300 x 300 DPI)

BMJ Open

The Responsive and Equitable Health Systems – Partnership on Non-Communicable Diseases (RESPOND) study: A mixed-methods, longitudinal, observational study on treatment seeking for hypertension in Malaysia and the Philippines

Journal:	*BMJ Open*
Manuscript ID	bmjopen-2018-024000.R1
Article Type:	Protocol
Date Submitted by the Author:	15-Jun-2018
Complete List of Authors:	Palafox, B; The London School of Hygiene and Tropical Medicine, Centre for Global Chronic Conditions<br>Seguin, Maureen; The London School of Hygiene and Tropical Medicine, The Centre for Global Chronic Conditions<br>McKee, Martin; The London School of Hygiene and Tropical Medicine, The Centre for Global Chronic Conditions<br>Dans, Antonio; University of the Philippines, College of Medicine<br>Yusoff, Khalid; UCSI University, Faculty of Medicine and Health Sciences; University Teknologi MARA, Centre for Translational Research & Epidemiology, Faculty of Medicine<br>Candari, Christine; University of the Philippines, College of Medicine<br>Idris, Khairuddin; University Putra Malaysia, Institute for Social Science Studies<br>Ismail, Johan; University Teknologi MARA, Centre for Translational Research & Epidemiology, Faculty of Medicine<br>Krauss, Steven; University Putra Malaysia, Institute for Social Science Studies<br>Lasco, Gideon; University of the Philippines Diliman, Department of Anthropology<br>Majid, Fadhlina; University Teknologi MARA, Centre for Translational Research & Epidemiology, Faculty of Medicine<br>Palileo-Villanueva, Lia; University of the Philippines, College of Medicine<br>Razak, Azlina; UCSI University, Faculty of Medicine and Health Sciences<br>Renedo, Alicia; The London School of Hygiene and Tropical Medicine, Social and Environmental Health Resarch<br>Balabanova, Dina; The London School of Hygiene and Tropical Medicine, The Centre for Global Chronic Conditions
**Primary Subject Heading**:	Public health
Secondary Subject Heading:	Health services research, Public health, Qualitative research
Keywords:	Hypertension < CARDIOLOGY, Health systems, Cardiovascular disease, Malaysia, Philippines

For peer review only

Title: The Responsive and Equitable Health Systems – Partnership on Non-Communicable Diseases (RESPOND) study: A mixed-methods, longitudinal, observational study on treatment seeking for hypertension in Malaysia and the Philippines

Corresponding author:
Palafox, Benjamin, London School of Hygiene & Tropical Medicine, 15-17 Tavistock Place, Kings Cross, London WC1H 9SH, benjamin.Palafox@lshtm.ac.uk, 02079588337

Full names, departments, institutions, city and country of all co-authors:
Palafox, Benjamin*, Centre for Global Chronic Conditions, London School of Hygiene and Tropical Medicine, London, UK

Seguin, Maureen L*, Centre for Global Chronic Conditions, London School of Hygiene and Tropical Medicine, London, UK

McKee, Martin, Centre for Global Chronic Conditions, London School of Hygiene and Tropical Medicine, London, UK

Dans, Antonio L, College of Medicine, University of the Philippines Manila, Manila, PH

Yusoff, Khalid, Faculty of Medicine and Health Sciences, UCSI University, Kuala Lumpur, MY and Centre for Translational Research & Epidemiology, Faculty of Medicine, University Teknologi MARA, Shah Alam, MY

Candari, Christine J, College of Medicine, University of the Philippines Manila, Manila, PH

Idris, Khairuddin, Institute for Social Science Studies, University Putra Malaysia, Serdang, MY

Ismail, Johan Rizwal, Centre for Translational Research & Epidemiology, Faculty of Medicine, University Teknologi MARA, Shah Alam, MY

Krauss, Steven Eric, Institute for Social Science Studies, University Putra Malaysia, Serdang, MY

Lasco, Gideon, Department of Anthropology, University of the Philippines Diliman, Quezon City, PH
Majid, Fadhlina A, Centre for Translational Research & Epidemiology, Faculty of Medicine, University Teknologi MARA, Shah Alam, MY

Palileo-Villanueva, Lia M, College of Medicine, University of the Philippines Manila, Manila, PH

Razak, Azlina A, Faculty of Medicine and Health Sciences, UCSI University, Kuala Lumpur, MY

Renedo, Alicia, Department of Social and Environmental Health Research, London School of Hygiene and Tropical Medicine, London, UK

For peer review only - http://bmjopen.bmj.com/site/about/guidelines.xhtml

Balabanova, Dina, Centre for Global Chronic Conditions, London School of Hygiene and Tropical Medicine, London, UK

*Benjamin Palafox and Maureen Seguin contributed equally to this paper

Keywords:
Hypertension, Health systems, Cardiovascular disease, Malaysia, Philippines

Word count excluding title page, abstract, references, figures and tables: 2952

Reference count: 34

ABBREVIATIONS

CVD cardiovascular disease, FGD focus group discussion, LMIC low- and middle-income country, RESPOND Responsive and Equitable Health Systems–Partnership on Non-Communicable Diseases, SARA Service Availability and Readiness Assessments

ABSTRACT

Introduction: Hypertension is a leading contributor to the global burden of disease. While safe and effective treatment exists, blood pressure control is poor in many countries, often reflecting barriers at the levels of health systems and services, as well as at the broader level of patients' socio-cultural contexts. This study examines how these interact to facilitate or hinder hypertension control, taking into account characteristics of service provision components and social contexts.

Methods and Analysis: The study, set in Malaysia and the Philippines, builds on two systematic reviews of barriers to effective hypertension management. People with hypertension (pre-existing and newly diagnosed) will be identified in poor households in 24-30 communities per country. Quantitative and qualitative methods will be used to examine their experiences of and pathways into seeking and obtaining care. These include two waves of household surveys of 20-25 participants per community 12-18 months apart, micro-costing exercises to assess the cost of illness (including costs due to health seeking activities and inability to work [5 per community]), preliminary and follow-up in-depth interviews and digital diaries with hypertensive adults over the course of a year (40 per country, employing an innovative mobile phone technology), focus group discussions with study participants, and structured assessments of health facilities (including formal and informal providers).

Ethics and Dissemination: Ethical approval has been granted by the Observational Research Ethics Committee at the London School of Hygiene and Tropical Medicine, and the Research Ethics Boards at the Universiti Putra Malaysia and the University of the Philippines Manila. The project team will disseminate findings and engage with a wide range of stakeholders to promote uptake and impact. Alongside publications in high-impact journals, dissemination activities include a comprehensive stakeholder analysis, engagement with traditional and social media and 'digital stories' co-produced with research participants.

For peer review only - http://bmjopen.bmj.com/site/about/guidelines.xhtml

STRENGTHS AND LIMITATIONS

- Our prospective cohort design study benefits from a solid evidence base consisting of two systematic reviews.
- A key strength of the proposed study is its mixed-method design including innovative mobile phone technology to enhance examination of patient pathways.
- We may encounter limited access to facilities, as facility staff may be reluctant to participate or fully disclose information due to for regulatory or commercial reasons.
- Participant attrition between the interview and follow-up interview and household survey phases may constitute a limitation to findings inferred from follow-up data.

BACKGROUND

Control of hypertension is an essential part of any strategy to reduce the burden of cardiovascular diseases (CVDs) worldwide.[1 2] Malaysia and the Philippines are countries where hypertension control is poor (Table 1), and many other low- and middle-income countries (LMICs) face similar challenges.[3 4] The poorest individuals in the lowest wealth quintile are especially disadvantaged, [4-7] with profound implications for their health and economic wellbeing.[8] As two recent systematic reviews showed, health systems and services barriers to effective management exist in countries at all levels of development but can be overcome.[9 10] However, a comprehensive approach will also take account of patients' socio-cultural contexts, exploring how they interact with health services to facilitate or hinder hypertension control.

Table 1: Treatment gap in hypertension among adults aged 35-70

Country	*Hypertension prevalence*	*% aware hypertensives*	*% of treated hypertensives*	*% of controlled hypertensives*	*% treated in wealthiest quintile*	*% treated in poorest quintile*
Canada (comparator)	*37.5*	*55.2*	*54.0*	*24.8*	*51.8*	*55.6*
Malaysia	*46.6*	*48.1*	*41.2*	*12.5*	*41.5*	*36.6*
Philippines	*51.2*	*54.5*	*46.1*	*13.5*	*64.5*	*34.1*

Source: PURE Study [4]

The core elements of hypertension management are well established, set out in the World Heart Federation Hypertension Roadmap and other guidelines for low-resource settings.[8 11] However, their specific application in each country must take account of the lived experiences of those with hypertension and frontline healthcare providers, exploring understanding of the condition and treatment, actual socio-cultural barriers to obtaining and adhering to treatment, and how barriers can be overcome. Our previous research in Malaysia and Colombia demonstrated the importance of local context.[12 13] Thus, the first objective of the Responsive and Equitable Health Systems – Partnership on Non-Communicable Diseases (RESPOND) project is to produce robust evidence on the barriers to effective hypertension management (i.e. at diagnosis, treatment initiation and adherence, and ultimately control) faced by poor households in Malaysia and the Philippines. This will be achieved through quantitative and qualitative observational methods in a longitudinal study design.

The experience of living with and managing hypertension is a dynamic process shaped by health services and people's social contexts and life circumstances.[13-16] Non-intrusive methods are needed to capture patients' perspectives of the changing complexities of seeking care over time. Thus, our second objective is to evaluate opportunities offered by mobile phone technology to study the lived

For peer review only - http://bmjopen.bmj.com/site/about/guidelines.xhtml

experience of hypertension and barriers people face in handling hypertension. Specifically, we will develop an innovative qualitative data collection method involving the use of real-time 'digital diaries' in both countries.

METHODS AND STUDY DESIGN

This is a prospective cohort design combining 3 quantitative and 3 qualitative elements to explore several research questions: How are patients with hypertension diagnosed? What pathways do patients follow through the key stages of care? What care is sought and received, and what are the economic and social costs? How does this differ by patient characteristic, and why? What barriers impede continuing access to care and medication? How does context affect experiences of care? How feasible and acceptable are 'digital diaries' for collecting longitudinal qualitative data on lived experiences with chronic illness?

In each country, the quantitative elements include: (a) 600 initial and follow-up household surveys (12-18 months apart) of hypertensive patients residing in 24-30 low-income rural and urban communities, (b) an embedded micro-costing study of 5 hypertensive participants in each community, and (c) approximately 200 structured facility assessments of hypertension care providers. The qualitative components are: (d) 40 initial and follow-up in-depth semi-structured interviews among a sub-sample of survey participants with (e) up to 40 digital diaries recorded by interviewees between interviews, and (f) 12 focus group discussions with hypertensive adults in 6 different communities. These elements are illustrated in Figure 1.

Initial and follow-up household surveys

In each country, 24-30 communities (*mukim* in Malaysia and *barangays* in the Philippines) will be selected, divided between urban and rural areas. In Malaysia, communities are in 4 peninsular states (Selangor, Kelantan, Perak and Johor). In the Philippines, 7 urban communities are in the City of Valenzuela in Metro Manila, and 8 urban and 15 rural communities in Quezon province. While these have been purposefully selected to facilitate access by researchers, we will randomly select adults living within poor households (low-income households qualifying for government subsides under the BR1M programme in Malaysia,[17] and the 4P programme in the Philippines[18]) to obtain a statistically representative sample.

Within selected households, eligible individuals are: a) aged 35-70 years at screening, b) living within households expected to remain at the current address/location for at least 18 months, and c) either a self-reported history of hypertension or identified as hypertensive during screening. Individuals with a self-reported history of either cancer and/or HIV will be excluded, as their health care use is likely to be atypical.

After informed consent is taken, recruited participants' blood pressure measurement will be taken using a digital sphygmomanometer following a standardised procedure. Hypertension is defined as blood pressure greater than or equal to 140/90 mm Hg or a self-reported history of hypertension.[19] Among all hypertensive members in eligible households, one will be invited to participate using a probability based method, such as the KISH, age-order or full enumeration methods.[20]

A questionnaire consisting of validated instruments, including the Demographic & Health Surveys,[21] WHO STEPS,[22] World Values Survey,[23] and the Living Conditions, Lifestyle and Health Survey,[24] will be administered to participants within their homes, collecting information on housing characteristics, including structure, amenities and household assets. A validated household asset-based wealth score, allowing within- and cross-country comparisons, will be calculated.[25 26] Detailed information from individuals and households will be collected, including age, gender, marital status, ethnicity,

For peer review only - http://bmjopen.bmj.com/site/about/guidelines.xhtml

education, literacy, occupation; hypertension-related care experiences in the past 12 months (including service utilisation, settings [formal and informal practitioners, including criteria for choosing], treatment and adherence, transportation to care, knowledge of hypertension, attitudes to hypertension and its treatment); personal and environmental tobacco exposure; socioeconomic factors including employment income, education, and psychosocial factors (social capital, attitudes and beliefs), and modifiable CVD risk factors for the 'non-laboratory' INTERHEART risk score.[27] Data on health care pathways will be subdivided into discrete stages along the journey, collecting details of experiences at each, including duration, location, processes undertaken, treatment provided, costs, and self-reported reasons for seeking care.[28] A second wave of interviews, using an adapted version of the first instrument, will be conducted on the same participants after 12 months.

Sample size calculation took account of many possible analyses. For example, the ability to detect an urban-rural difference in treatment of hypertension in a middle-income country with $\alpha=0.05$ and power of 0.8 (two-tailed), which one recent study suggested was as large as 13 percentage points (42% urban, 28% rural),[3] would require a sample of 364 individuals with hypertension.

Micro-costing study

The micro-costing study assesses the economic burden of hypertension on poor households. All who consent to participate in the household survey and are aware of their hypertension at baseline will be eligible to participate. Five in each community will be selected randomly to provide micro-costing data. They will be asked additional questions related to household income and expenditure, health expenditure related to hypertension management, health financing (including insurance coverage) and coping strategies (labour substitution for ill person and/or caretaker, use of savings, changing consumption patterns, sale of assets, borrowing, other strategies). If participants cannot provide the requested information (e.g. because they are not involved in the management of household finances), they will be asked to consult a knowledgeable household member. The same information will be collected at baseline and follow-up.

Facilities assessment

Health system assessments will be undertaken contemporaneously with the second round of household surveys. These will provide 'focused snapshots' of the hypertension care available to communities. Facilities included are those where patients receive care for hypertension within the 6 communities in both countries where focus group discussions will be conducted (see below). They will be identified according to participant-led utility of health care providers, encompassing different levels of public, private, not-for-profit hospitals and clinics, complementary care providers, traditional and alternative healers, community health workers, outreach programmes/missions, retail pharmacies, drug stores or dispensaries, general retailers, and/or mobile vendors. Within each of the 6 communities in both countries, a minimum of 2-3 providers of each type within the community will be included, generating up to 200 assessments across a very diverse range of providers.

Trained data collectors will use a structured facility questionnaire informed by the Service Availability and Readiness Assessments (SARA) developed by the World Health Organisation[29] to characterise care in selected facilities, including infrastructure and work conditions; human resources; equipment, medicines and supplies; written information and educational materials.[30] Questionnaires will also examine providers' practices, perspectives and experiences, and provide space for data collectors to provide free text information on their impressions and comments on the respondent, facility and any other observations.

In-depth qualitative interviews

For peer review only - http://bmjopen.bmj.com/site/about/guidelines.xhtml

Initial and follow-up in-depth qualitative interviews will be conducted with a total of 80 participants (40 each in Malaysia and the Philippines). These will be purposively sampled from the household survey participants. The local teams will seek to recruit a balanced sample by sex, age, stage of treatment process, rural-urban locality, and health complexity. Each participant will be interviewed shortly after completion of the household survey and again after 12-18 months have passed. Interviews will be conducted by the local research team and will be audio-recorded and transcribed verbatim for analysis. Information about hypertension services will be provided to all participants after the interview.

The interview will be conversational and explore participants' views on hypertension, including treatment and care, as well as their own personal experiences of living with the condition and of accessing and using various types of healthcare services, including traditional and alternative medicine. We will attempt to understand people's conception of hypertension (including its diagnosis and treatment) and lived healthcare experiences and trajectories in relation to their wider socio-cultural and environmental contexts (e.g. work, community, and family relationships). Follow-up interviews will explore changes over time and issues raised in the first interview and during digital diary recording (see below). To address our secondary methodological objective, we will add additional questions for those who have completed digital diaries in order to explore their experiences of using the mobile-phone diary technology and to assess the overall feasibility of this qualitative data collection method.

Digital diaries

All interview participants will be invited to complete optional digital diaries (via mobile phones) between the initial and follow-up interviews, yielding a maximum of 80 digital diaries from both countries. Those who consent but do not have a mobile phone will be provided with one (along with mobile phone credit) from the study team to allow the recording of their digital diary. Those who already possess mobile phones will only receive mobile phone credit. The participants will be trained on how to submit diary entries, provided with a guidance leaflet to keep after the training, and continually engaged for in-depth information on patient experience.

Diary participants will be encouraged to record their experiences of living with hypertension, the barriers to treatment and control of hypertension they and their families face and, crucially, their view of feasible solutions. Participants will be able to submit audio (spoken), written (text messages), and visual (photo, video) material via mobile phones. Study team members will explain to participants that diary recording is totally voluntary, and that they can record as much as they prefer. They will also discuss with them whether they would be happy to receive probes via text message from study members in some occasions. Through such probing, respondents can reflect upon and reappraise their own experiences with hypertension and use of services, and thus improve the depth of the data. If participants do not submit any entries in the first two months of enrolling into the study, they will be replaced with another participant.[31]

Focus group discussions

Twelve focus group discussions (FGDs) with study participants will be held after the follow-up interviews have been completed, comprising six each from the Philippines and Malaysia. We aim for between 7 and 10 participants per FGD, yielding a total FGD sample between 84 and 120. At least one FGD will be conducted exclusively with participants who completed digital diaries. The study team will strive to match individuals with other FGD participants by age group and sex.

The focus groups offer an opportunity to access shared and conflicting aspects of the participant's health beliefs and experiences and social processes that shape individual decision-making about

For peer review only - http://bmjopen.bmj.com/site/about/guidelines.xhtml

treatment, accessing services etc. The discussion will focus on trajectories of care and participants' own prioritisation of issues and solutions.

Patient and Public Involvement

The development of our research questions and study design was based on the premise that effective management of hypertension requires patient-centred health systems that respond to patient needs, health seeking behaviour and preferences, which often vary among and within countries. We have engaged with stakeholders and community representatives, including patient groups, during the project inception phase to identify major challenges faced by the poor and hard-to-reach communities, and to understand local culture and how it may affect the study. We will maintain this engagement throughout field work and analysis to ensure that we can respond to emerging changes in the field and allow adaptation in real time. In line with our patient-centred approach, several key outputs will be co-produced jointly between communities and researchers. Some of these outputs were designed explicitly to engage the public through digital stories, policy briefings, webcasts, and interactive panels.

ANALYSIS

Quantitative components

To describe pathways of care, we will draw on methods used previously to examine the unique sequences of care-seeking behaviours taken by hypertensive individuals to manage their condition.[28] The resulting data will be used to analyse the determinants of hypertension detection, treatment and control using appropriate regression models. We will also use multi-level models that nest the experience of the individual within their community and health systems contexts, to obtain community-adjusted estimates.

The household economic burden of hypertension will be assessed by summing all direct and indirect costs incurred by households related to hypertension over the preceding 12 months. Indirect costs will be estimated using the human capital model,[32] accounting for lost productivity due to morbidity and premature mortality. Morbidity costs are defined as lost income due to disability and inability to work as a result of hypertension and/or its consequences, calculated by multiplying total of days off work by a context-appropriate valuation of average gross daily earnings. Mortality costs are defined as lost income attributable to hypertension-related premature mortality, multiplying years of life lost by a context-appropriate valuation of annual net income. Years of life lost will be calculated using the Global Burden of Disease methods.[33] Catastrophic health payment will be defined as occurring when health expenditures exceed 40% of a household's non-food expenditures or capacity to pay for services, in the 12 months prior to the interview; a range of thresholds will be used for sensitivity analyses.

Qualitative components

Analysis of qualitative data generated via interviews, digital diaries, and FGDs will be conducted by local researchers with support by the London team. The analysis will focus on how participants (and carers) make sense of hypertension and the experience of living with and managing the condition and accessing care. It will also examine the temporal ordering of events in care seeking and management of hypertension, to understand links between actions and consequences over time and across contexts. A combined deductive and inductive approach will guide the analysis,[34] with the process reviewed and refined iteratively. A coding framework will be developed using Nvivo 11 qualitative analysis software, followed by an analytical process of identifying, comparing, and contrasting increasingly abstract themes, within and across countries.

Data management

For peer review only - http://bmjopen.bmj.com/site/about/guidelines.xhtml

All digital data will be anonymised and transferred to secure servers. The data will be accessible to project members in the UK, Malaysia, and the Philippines via LSHTM's secure/password protected web portal. User authentication and encryption will be applied throughout. Laptops and data sticks used in data collection will be encrypted, and institutional networks are protected using user authentication. Any printed material used during transcription will be stored in a locked cabinet and destroyed when no longer needed.

Ethics and dissemination

Ethical approval has been granted by the LSHTM Observational Research Ethics Committee and the Research Ethics Boards at the Universiti Putra Malaysia and the University of the Philippines Manila. Our knowledge dissemination strategy includes publication of academic articles in high-impact journals, engagement with key stakeholders informed by a stakeholder analysis, and contributions to traditional and social media.

DISCUSSION

The RESPOND project is particularly appropriate to assess the health needs of poor populations in Malaysia and the Philippines. The economic and treatment burden of hypertension is likely to disproportionately affect poor populations who suffer multiple disadvantage and substantial disease burden, both infectious and non-communicable. Means to address this issue, and the growing NCD burden more broadly, is now an important policy priority. Evidence from the RESPOND study will promote essential insights to design and implement contextually appropriate initiatives.

While many of the findings from the RESPOND project will be specific to the two countries, some will be more widely applicable. The project will also inform the development of methods that can be used in other low- and middle-income countries at a similar stage of the epidemiological transition, with relatively under-resourced health systems where patient-centred approaches focused on the needs of the poor can be further enhanced. This is facilitated by our framing of the research questions and design within the global literature on NCD, ensuring possibilities for comparisons with other settings, supporting exposure of the researchers involved to international debates and engagement with dissemination activities at global and national levels.

ACKNOWLEDGMENTS

The authors would like to thank the Wellcome Trust/Newton Fund-MRC Humanities & Social Science Collaborative Award scheme for providing funding for this research.

FUNDING

This study is supported by a grant from the Wellcome Trust/Newton Fund-MRC Humanities & Social Science Collaborative Award scheme (200346/Z/15/Z).

CONTRIBUTORS

BP, MM, DB, AAR, ALD and KY conceptualised the study. BP, MS, MM, ALD, KY, CC, KI, JRI, SEK, GL, FAM, LMPV, AAR, AR, and DB contributed to the development of study design. BP, MS, MM, ALD, KY, CC, KI, JRI, SEK, GL, FAM, LMPV, AAR, AR, and DB read, edited, and approved the final version. MS and BP contributed equally to the first draft of the manuscript.

COMPETING INTERESTS

For peer review only - http://bmjopen.bmj.com/site/about/guidelines.xhtml

For peer review only

ALD, KY, JRI, FAM and LMPV have been and/or are currently involved in clinical trials of antihypertensive medications that receive some funding from industry.

Figure 1: Study elements in Malaysia and the Philippines over 18 months

REFERENCES

1. GBD 2016 SDG Collaborators. Measuring progress and projecting attainment on the basis of past trends of the health-related Sustainable Development Goals in 188 countries: an analysis from the Global Burden of Disease Study 2016. *Lancet (London, England)* 2017;390(10100):1423-59. doi: 10.1016/s0140-6736(17)32336-x [published Online First: 2017/09/17]
2. GBD 2016 Risk Factors Collaborators. Global, regional, and national comparative risk assessment of 84 behavioural, environmental and occupational, and metabolic risks or clusters of risks, 1990-2016: a systematic analysis for the Global Burden of Disease Study 2016. *Lancet (London, England)* 2017;390(10100):1345-422. doi: 10.1016/s0140-6736(17)32366-8 [published Online First: 2017/09/19]
3. Chow CK, Teo KK, Rangarajan S, et al. Prevalence, awareness, treatment, and control of hypertension in rural and urban communities in high-, middle-, and low-income countries. *JAMA* 2013;310(9):959-68. doi: 10.1001/jama.2013.184182
4. Palafox B, McKee M, Balabanova D, et al. Wealth and cardiovascular health: a cross-sectional study of wealth-related inequalities in the awareness, treatment and control of hypertension in high-, middle- and low-income countries. *International Journal for Equity in Health* 2016;11(199) doi: 10.1186/s12939-016-0478-6
5. Lloyd-Sherlock P, Beard J, Minicuci N, et al. Hypertension among older adults in low- and middle-income countries: prevalence, awareness and control. *International Journal of Epidemiology* 2014 doi: 10.1093/ije/dyt215
6. Mendez MA, Cooper R, Wilks R, et al. Income, education, and blood pressure in adults in Jamaica, a middle-income developing country. *Int J Epidemiol* 2003;32(3):400-8.
7. Moser KA, Agrawal S, Davey Smith G, et al. Socio-Demographic Inequalities in the Prevalence, Diagnosis and Management of Hypertension in India: Analysis of Nationally-Representative Survey Data. *PLoS ONE* 2014;9(1):e86043. doi: 10.1371/journal.pone.0086043
8. Adler AJ, Prabhakaran D, Bovet P, et al. Reducing Cardiovascular Mortality Through Prevention and Management of Raised Blood Pressure: A World Health Federation Roadmap. *Global Heart* 2015;10(2):111-22. doi: 10.1016/j.gheart.2015.04.006
9. Khatib R, Schwalm J-D, Yusuf S, et al. Patient and Healthcare Provider Barriers to Hypertension Awareness, Treatment and Follow Up: A Systematic Review and Meta-Analysis of Qualitative and Quantitative Studies. *PLoS ONE* 2014;9(1):e84238. doi: 10.1371/journal.pone.0084238
10. Maimaris W, Paty J, Perel P, et al. The influence of health systems on hypertension awareness, treatment, and control: a systematic literature review. *PLoS medicine* 2013;10(7):e1001490. doi: 10.1371/journal.pmed.1001490
11. Modesti PA, Agostoni P, Agyemang C, et al. Cardiovascular risk assessment in low-resource settings: a consensus document of the European Society of Hypertension Working Group on Hypertension and Cardiovascular Risk in Low Resource Settings. *J Hypertens* 2014;32(5):951-60. doi: 10.1097/hjh.0000000000000125 [published Online First: 2014/03/01]

For peer review only - http://bmjopen.bmj.com/site/about/guidelines.xhtml

12. Risso-Gill I, Balabanova D, Majid F, et al. Understanding the modifiable health systems barriers to hypertension management in Malaysia: a multi-method health systems appraisal approach. *BMC Health Serv Res* 2015;15:254. doi: 10.1186/s12913-015-0916-y
13. Legido-Quigley H, Camacho Lopez PA, Balabanova D, et al. Patients' knowledge, attitudes, behaviour and health care experiences on the prevention, detection, management and control of hypertension in Colombia: a qualitative study. *PLoS One* 2015;10(4):e0122112. doi: 10.1371/journal.pone.0122112
14. Morgan M, Watkins CJ. Managing hypertension: beliefs and responses to medication among cultural groups. *Sociology of Health & Illness* 1988;10(4):561-78. doi: 10.1111/1467-9566.ep10837256
15. Marshall IJ, Wolfe CDA, McKevitt C. Lay perspectives on hypertension and drug adherence: systematic review of qualitative research. *BMJ : British Medical Journal* 2012;345 doi: 10.1136/bmj.e3953
16. Higginbottom GM. 'Pressure of life': ethnicity as a mediating factor in mid-life and older peoples' experience of high blood pressure. *Sociol Health Illn* 2006;28(5):583-610. doi: 10.1111/j.1467-9566.2006.00508.x [published Online First: 2006/08/17]
17. Kamaruddin R, Othman AA, Denan Z. Government Sincere Initiatives or Political Motives of 1Malaysia Peoples' Aid: Using Structural Equation Modeling. *Procedia - Social and Behavioral Sciences* 2013;105(Supplement C):715-22. doi: https://doi.org/10.1016/j.sbspro.2013.11.074
18. Republic of the Philippines. Pantawid Pamilyang Pilipino Program (4P) Manila: Government of the Philippines; 2017 [Available from: http://www.officialgazette.gov.ph/programs/conditional-cash-transfer/ accessed 10 November 2017.
19. Gee ME, Campbell N, Sarrafzadegan N, et al. Standards for the uniform reporting of hypertension in adults using population survey data: recommendations from the World Hypertension League Expert Committee. *Journal of clinical hypertension* 2014;16(11):773-81. doi: 10.1111/jch.12387
20. Gaziano C. Comparative Analysis of Within-Household Respondent Selection Techniques. *Public Opinion Quarterly* 2005;69(1):124-57. doi: 10.1093/poq/nfi006
21. USAID. Demographic and Health Surveys 2017 [Available from: https://dhsprogram.com/ accessed 10th October 2017.
22. World Health Organization. WHO STEPS surveillance manual: the WHO STEPwise approach to chronic disease risk factor surveillance. Geneva: WHO 2005.
23. Inglehart R, Puranen B, Pettersson T, et al. The World Values Survey. 2005
24. Balabanova D, Roberts B, Richardson E, et al. Health care reform in the former Soviet Union: beyond the transition. *Health services research* 2012;47(2):840-64. doi: 10.1111/j.1475-6773.2011.01323.x [published Online First: 2011/11/19]
25. Filmer D, Pritchett LH. Estimating wealth effects without expenditure data--or tears: an application to educational enrollments in states of India. *Demography* 2001;38(1):115-32. [published Online First: 2001/03/03]
26. Vyas S, Kumaranayake L. Constructing socio-economic status indices: how to use principal components analysis. *Health Policy Plan* 2006;21(6):459-68. doi: 10.1093/heapol/czl029
27. Yusuf S, Hawken S, Ounpuu S, et al. Effect of potentially modifiable risk factors associated with myocardial infarction in 52 countries (the INTERHEART study): case-

For peer review only - http://bmjopen.bmj.com/site/about/guidelines.xhtml

control study. *Lancet* 2004;364(9438):937-52. doi: 10.1016/s0140-6736(04)17018-9 [published Online First: 2004/09/15]

28. Haenssgen MJ, Ariana P. Healthcare access: A sequence-sensitive approach. *SSM - Population Health* 2017;3:37-47. doi: http://dx.doi.org/10.1016/j.ssmph.2016.11.008
29. Organization WH. Service availability and readiness assessment (SARA): an annual monitoring system for service delivery: reference manual. Geneva: WHO 2013.
30. O'Neill K, Takane M, Sheffel A, et al. Monitoring service delivery for universal health coverage: the Service Availability and Readiness Assessment. *Bulletin of the World Health Organization* 2013;91:923-31.
31. Patton MQ. Qualitative research and evaluation methods. 2 ed. London: Sage Publications 2002.
32. Pritchard C, Sculpher M. Productivity Costs: Principles and Practice in Economic Evaluation. London: Office of Health Economics 2000.
33. Murray CJL, Lopez AD. Measuring the Global Burden of Disease. *New England Journal of Medicine* 2013;369(5):448-57. doi: 10.1056/NEJMra1201534
34. Braun V, Clarke V. Using thematic analysis in psychology. *Qualitative Research in Psychology* 2006;3(2):77-101.

For peer review only

For peer review only - http://bmjopen.bmj.com/site/about/guidelines.xhtml

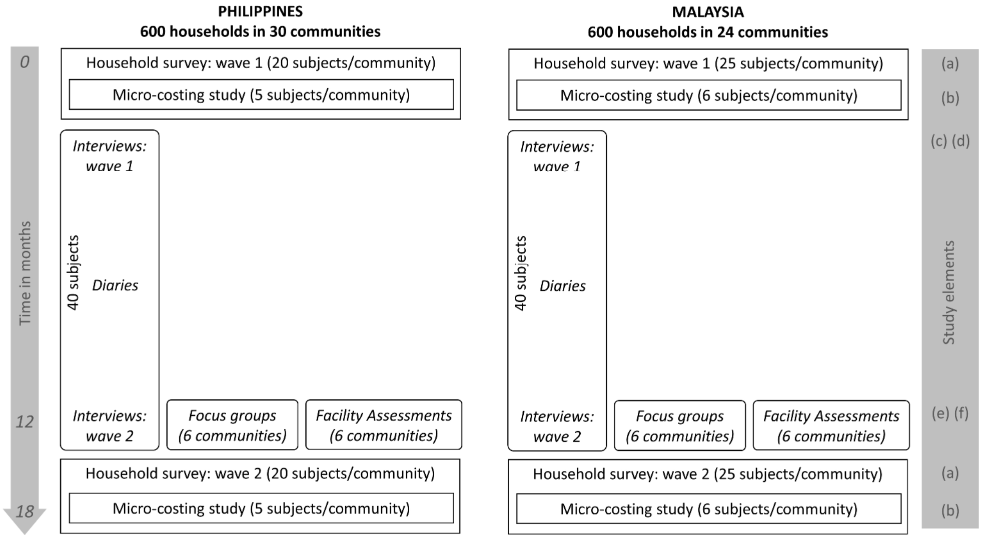

Study elements in Malaysia and the Philippines over 18 months

338x190mm (300 x 300 DPI)
