# Supplementary material for: Responsive and Equitable Health Systems—Partnership on Non-Communicable Diseases (RESPOND) study: a mixed-methods, longitudinal, observational study on treatment seeking for hypertension in Malaysia and the Philippines
Source: BMJ Open. 2018 Jul 30;8(7):e024000. doi: 10.1136/bmjopen-2018-024000 (PMC6067392; doi:10.1136/bmjopen-2018-024000)
Supplement: Reviewer comments [file bmjopen-2018-024000.reviewer_comments.pdf]

PEER REVIEW HISTORY

BMJ Open publishes all reviews undertaken for accepted manuscripts. Reviewers are asked to complete a checklist review form (**http://bmjopen.bmj.com/site/about/resources/checklist.pdf**) and are provided with free text boxes to elaborate on their assessment. These free text comments are reproduced below.

ARTICLE DETAILS

**TITLE (PROVISIONAL)**	The Responsive and Equitable Health Systems – Partnership on Non-Communicable Diseases (RESPOND) study: A mixed-methods, longitudinal, observational study on treatment seeking for hypertension in Malaysia and the Philippines
**AUTHORS**	Palafox, B; Seguin, Maureen; McKee, Martin; Dans, Antonio; Yusoff, Khalid; Candari, Christine; Idris, Khairuddin; Ismail, Johan; Krauss, Steven; Lasco, Gideon; Majid, Fadhlina; Palileo-Villanueva, Lia; Razak, Azlina; Renedo, Alicia; Balabanova, Dina

This article was not externally reviewed at BMJ Open. Protocols that have been independently assessed prior to submission to BMJ Open are usually fast-tracked to publication on the grounds that further substantial changes will not be possible. This independent assessment will usually be external, independent review for both a substantial grant award from a non-commercial or government funder and ethics approval.
